# Interaction between Work and Metabolic Syndrome: A Population-Based Cross-Sectional Study

**DOI:** 10.3390/healthcare10030544

**Published:** 2022-03-15

**Authors:** Amália Ivine Costa Santana, Magno Conceição das Merces, Marcio Costa de Souza, Bruno Gil de Carvalho Lima, Maria José Quina Galdino, Nuno Damácio de Carvalho Félix, Lucelia Batista Neves Cunha Magalhães, Julita Maria Freitas Coelho, Paulo José Bastos Barbosa, Érica Velasco Dias Gomes, Rodrigo Fernandes Weyll Pimentel, Anderson Reis de Sousa, Márcia Aparecida Ferreira de Oliveira, Aline Macêdo de Queiroz, Raíssa Millena Silva Florencio, Jorge Lopes Cavalcante Neto, Antonio Marcos Tosoli Gomes, Thadeu Borges Souza Santos, Silvana Lima Vieira, Danilo Guimarães de Sousa, Priscila Cristina da Silva Thiengo de Andrade, Isolda Prado de Negreiros Nogueira Maduro, Sandra Lúcia Fernandes, Kairo Silvestre Meneses Damasceno, Dandara Almeida Reis da Silva, Argemiro D’Oliveira Júnior

**Affiliations:** 1 Health Sciences Postgraduate Program, School of Medicine, Federal University of Bahia (UFBA), Salvador 40026-010, Bahia, Brazil; amalia0807@gmail.com (A.I.C.S.); rodrigo.pimentel@ebserh.gov.br (R.F.W.P.); kairodamasceno@hotmail.com (K.S.M.D.); argemiro@ufba.br (A.D.J.); 2 Department of Life Sciences, State University of Bahia (UNEB), Salvador 41150-000, Bahia, Brazil; mcsouza@uneb.br (M.C.d.S.); julitamaria@gmail.com (J.M.F.C.); pjbarbosa@uneb.br (P.J.B.B.); enfa.ericavelasco@gmail.com (É.V.D.G.); jlcavalcante@uneb.br (J.L.C.N.); tbssantos@uneb.br (T.B.S.S.); slvieira@uneb.br (S.L.V.); danilogs26@gmail.com (D.G.d.S.); darsilva@uneb.br (D.A.R.d.S.); 3 FTC University Center (UniFTC), Federal University of Bahia (UFBA), Salvador 41741-590, Bahia, Brazil; brunogil@doctor.com (B.G.d.C.L.); luceliamagalhaes@terra.com.br (L.B.N.C.M.); 4 Department of Pathology and Forensic Medicine, School of Medicine, Federal University of Bahia (UFBA), Salvador 40026-010, Bahia, Brazil; 5 Department of Nursing, State University of Northern Paraná (UENP), Bandeirantes 86360-000, Paraná, Brazil; mariagaldino@uenp.edu.br; 6 Health Sciences Center, Federal University of Recôncavo of Bahia (UFRB), Santo Antônio de Jesus 44574-490, Bahia, Brazil; nunofelix@ufrb.edu.br; 7 Department of Family Health, School of Medicine, Federal University of Bahia (UFBA), Salvador 40026-010, Bahia, Brazil; 8 Brazilian Association of Nutrition (ABRAN), Catanduva 15801-150, São Paulo, Brazil; isoldaprado@yahoo.com.br (I.P.d.N.N.M.); sandranut@yahoo.com.br (S.L.F.); 9 School of Nursing, Federal University of Bahia (UFBA), Salvador 40231-300, Bahia, Brazil; anderson.sousa@ufba.br; 10 Department of Maternal-Child and Psychiatric Nursing, School of Nursing, University of São Paulo (USP), São Paulo 05403-000, São Paulo, Brazil; marciaap@usp.br; 11 School of Nursing, Federal University of Pará (UFPA), Belém 66075-110, Pará, Brazil; alinemacedo@ufpa.br (A.M.d.Q.); raissaflorencio@yahoo.com.br (R.M.S.F.); 12 School of Nursing, State University of Rio de Janeiro (UERJ), Rio de Janeiro 20551-030, Rio de Janeiro, Brazil; mtosoli@gmail.com (A.M.T.G.); profprithiengo@gmail.com (P.C.d.S.T.d.A.); 13 Department of Clinical Nutrition, State University of Amazonas (UEA), Manaus 69850-000, Amazonas, Brazil

**Keywords:** epidemiological studies, metabolic syndrome, professional burnout, worker’s health, primary health care

## Abstract

Metabolic syndrome (MS) is a clinical condition and a relevant risk factor in the development of cardiovascular diseases; it occurs as a result of lifestyle factors, e.g., work. The aim of this research was to estimate the interaction between work and MS among primary health care (PHC) nursing professionals in the state of Bahia, Brazil. A sectional multicentered study carried out in 43 municipalities in Bahia, whose study population consisted of nursing professionals. The exposure variables were occupation, professional exhaustion, and working time, and the outcome variable was MS. Interaction measures based on the additivity criteria were verified by calculating the excess risks due to the interactions and according to the proportion of cases attributed to the interactions and the synergy index. The global MS prevalence is 24.4%. There was a greater magnitude in the exposure group regarding the three investigated factors (average level occupation, professional exhaustion, and working time in PHC for more than 5 years), reaching an occurrence of 44.9% when compared to the prevalence of 13.1% in the non-exposure group (academic education, without professional burnout, and working time in PHC for up to 5 years). The study’s findings showed a synergistic interaction of work aspects for MS occurrence among PHC nursing professionals.

## 1. Introduction

Increasing morbidity and mortality rates trends, due to non-communicable chronic diseases (NCDs), are considered public health issues [1]; this has resulted in quality of life loses for patients, as well as considerable economic burdens at all health system levels. The causes of NCDs include metabolic and cardiocerebrovascular disorders, whose increases are directly influenced by metabolic syndrome (MS) [2].

MS is as a group of risk factors that could lead to the development of diabetes and cardiovascular diseases. It causes disturbances in body homeostasis, including glucose and lipid metabolism, changes in blood pressure, visceral fat deposition, and pro-inflammatory states. These concomitant disturbances may increase negative health outcomes [3]. Epidemiological surveys show a prevalence between 38.4% and 44.0% in the adult Brazilian population, who are influenced by genetic susceptibilities and individual behaviors, but mainly by social health determinants, such as work [2].

In this regard, the literature shows that work aspects are associated with MS [4,5], making it plausible to assume that the occupational context is capable of being an exposure factor in MS development. The following three variables have been listed as capable of stimulating MS: occupation, professional exhaustion, and working time. Findings from previous scientific investigations show them as risk factors through social vulnerability, disturbance of neuroendocrine activity, and allostatic load maintenance, respectively [4,6]. However, we have not found studies evaluating the interactions among these factors.

An interaction analysis is a powerful tool to clarify the paths of the health–disease process as it shows the risk factors that take place in the same causal mechanism. This analysis model assesses the occurrence behavior of a disease based on a combination of factors, not merely on the sum of their individual effects; that is, this model identifies cases that exist only by the combined effects of the investigated factors [7]. Despite the theoretical and methodological points of view that support the model, such an analysis is important from the point of view of public health, as it indicates that the elimination of a risk factor within the same causal mechanism can significantly reduce the risk of occurrence of disease in populations.

After overcoming the single-cause prerogative, the need for epidemiological approaches is reasonable, especially ones that incorporates analysis techniques toward the complexity of diseases. Based on this assumption and considering the lack of studies in the literature, this study estimates the interaction between work (occupation, professional exhaustion and working time) and MS in primary health care (PHC) nursing professionals in Bahia, Brazil.

## 2. Materials and Methods

### 2.1. Study Design and Location

This is a cross-sectional, population-based multicenter analysis linked to a large project, carried out in 43 municipalities in Bahia. 

### 2.2. Sample and Eligibility Criteria

The study population consisted of PHC nursing professionals, for purposes of random and equiprobable calculations; the sample was stratified by health mesoregions and later by municipal units. Using a simple random sample, 10% of the municipalities in each mesoregion were drawn. All 1195 PHC nursing professionals in the selected [4,8,9] municipalities were considered eligible. Prior to data collection, a pilot study was carried out with a similar population (n = 30). Considering the outcome, the design effect (α error at 0.05, 90% power, 1:1 ratio), possibility of refusals, and other losses, this procedure allowed us to calculate the sample of 1114 individuals, ensuring a representative sample of the PHC nursing workforce in Bahia, Brazil.

The criteria were the following: PHC nursing professionals over 18 years, who were working without restrictions at the time of data collection, having at least 6 months in the health unit, and who were available for blood collection for diagnosis of metabolic syndrome. Professionals who performed strictly administrative activities, pregnant women with self-reported diagnoses of “burnout syndrome”, common mental disorders (depression or anxiety), liver cirrhosis, illicit drug users, or alcohol abuse were not part of the sample [4,8,9]. 

### 2.3. Data Collect

Data collection took place between 2017 and 2018 through visits to the health units where the selected nursing professionals worked. For self-reported information and anthropometric measurements, research instruments were used, according to the literature review, and applied by previously trained interviewers, who had questions related to sociodemographic characteristics, labor characteristics, lifestyle, and human biology. The collection of blood samples after a 12-h fasting was performed and analyzed by only one laboratory and considered as the reference in each of the cities studied.

### 2.4. Outcome Variable

The outcome variable was MS and dichotomized into yes = 1/no = 0, based on diagnostic confirmation in the criteria of the I Brazilian Guidelines on the Diagnosis and Treatment of Metabolic Syndrome and the *National Cholesterol Education Program’s Adult Treatment Panel III* (3): abdominal obesity (men: ≥102 cm; women: ≥88 cm); triglycerides (≥150 mg/dL); high density lipoprotein (HDL) cholesterol (men: <40; women: <50 or pharmacological treatment for dyslipidemia); blood pressure (systolic: ≥130 mmHg and diastolic: ≥85 mmHg or use of antihypertensive drugs); fasting glucose (≥110 mg/dL or treatment for diabetes mellitus). Conventional enzymatic and colorimetric laboratory techniques were used to obtain serum dosages of fasting glucose, HDL cholesterol, and triglycerides. The professional who had three positive parameters was considered as a case of MS [3].

Abdominal obesity was verified by measuring the waist circumference (WC), whose values reflect a reliable indicator of visceral adipose tissue [10]. WC was measured in a private office, protecting the professional’s privacy, with the individual in an anatomical position, relaxed abdomen, using an inelastic measuring tape with a scale in centimeters of the ISP^®^ brand (Wiso, Santa Tereza, Paraná, Brazil), in duplicate and without compressing the skin. The midpoint of the distance between the lower edge of the rib cage and the iliac bone was used as reference body landmarks [11]. 

Blood pressure was measured following the recommendations of the 7th Brazilian Guideline on Arterial Hypertension, which provides guidance on the treatment of arterial hypertension and the prevention of its possible complications [12,13]. In this procedure, a Littmann^®^ stethoscope (classic model III, 3M, St. Paul, MN, USA) and a BD^®^ aneroid sphygmomanometer (adult size cuff, Franklin Lakes, NJ, USA) were used and previously calibrated. Two measurements were taken on the left upper limb, unclothed, with the nursing professional sitting after 5 min of rest (bladder emptying was requested), lower limbs uncrossed, using the average of the previous two measurements, with intervals of 5 min between them.

### 2.5. Exposure Variables

The exposure variables considered for the interaction analysis were occupation, professional exhaustion, and working time. The exposure variable ‘occupation’ was measured from the block on labor characteristics using a question from the research instrument entitled “professional category”, dichotomized into nurse = 0/auxiliary or nursing technician = 1. The variable reflected the occupation that the professional performed in the health unit and not necessarily the level of professional training. 

Professional burnout was measured by using the *Maslach Burnout Inventory–Human Services Survey (MBI–HSS)* translated and adapted to Brazilian Portuguese [14]. It has 22 items in the *Likert-type scale* (1—never, 2—rarely, 3—sometimes, 4—frequently, 5—always) related to work aspects with the following dimensions: emotional exhaustion (nine questions), depersonalization/skepticism (five questions), and reduced professional fulfillment/personal effectiveness (eight questions). Each dimension is rated as low, medium, and high. Regarding emotional exhaustion, the low score is ≤14; average is between 15 and 24; and high ≥ 25. For the depersonalization/skepticism dimension, a score of ≤3 means low, between 4 and 9 is medium, and ≥10 is high. Finally, for reduced professional fulfillment/personal effectiveness, scores ≥ 40 indicate a low index, between 33 and 39 points is a medium index, and ≤32 a high index; the logic of the *score* for this dimension is the opposite of the referred one. For purposes of dichotomizing the variable, which is a necessary procedure for the interaction analysis, we considered Ramirez’s criterion [15]: high scores in the dimensions of emotional exhaustion and depersonalization/skepticism and low scores for reduced professional fulfillment/personal effectiveness were classified as a case of professional burnout (yes = 1); on the other hand, low scores in the dimensions of emotional exhaustion and depersonalization/skepticism and high scores for reduced professional fulfillment/personal effectiveness, such as the absence of professional burnout (no = 0).

The working time was extracted from the research instrument based on labor characteristics whose question involved “working time in primary health care”. The variable was dichotomized into “up to 5 years of work” in APS = 0 and “more than 5 years of work” in APS = 1. The study hypothesis assumes synergistic interaction between the variables ‘work’ and MS.

### 2.6. Data Analysis

Data typing and processing were performed on Statistic Package for Social Sciences—SPSS, version 22.0 (IBM Corporation, New York, NY, USA) for Windows. Data analyses were made on STATA for Windows, version 14.0 (StataCorp, College Station, TX, USA), in the Laboratory for Teaching, Research, and Extension in Collective Health (LEPESC) of the State University of Bahia (UNEB), Brazil. Initially, a descriptive analysis of the variables, in terms of absolute and relative frequencies, was performed, as well as global MS prevalence. In the second stage of the analysis, we sought to evidence the association between work characteristics and MS. Therefore, a bivariate analysis was carried out to verify the association between the variables of interest to the study and the outcome. Prevalence ratios (PR), 95% confidence intervals (95% CIs), and *p* values were calculated using the chi-square test, whose statistical significance criteria were adopted to obtain values ≤ 0.05.

The interaction analysis involved the construction of dummy variables to establish exposures, where 0 represented no exposure for the variable considered and 1 the exposure status. The groups established were: no exposure to any of the factors = nurses, without professional burnout, working time in PHC up to 5 years (R000); independent exhibitions = Nursing Assistants/Technicians, without professional exhaustion, working time in PHC up to 5 years (R100); Nurses, with professional exhaustion, working time in PHC up to 5 years (R010); Nurses, without professional exhaustion, working time in PHC for more than 5 years (R001); and combined exposures = nursing assistants/technicians, with professional exhaustion, working time in PHC for up to 5 years (R110); nursing assistants/technicians, without professional burnout, working time in PHC for more than 5 years (R101); nurses, with professional exhaustion, working time in PHC for more than 5 years (R011); nursing assistants/technicians, with professional exhaustion, working time in PHC for more than 5 years (R111).

Interaction measures [7] based on additivity criteria were verified by calculating the excess risk due to the interaction

RERI = PR11 − PR01 − PR10 + 1

which quantifies the distance from the null value; by the proportion of cases attributed to the interaction

$$\mathrm{AP}=\frac{\left(\mathrm{PR}11-\mathrm{PR}01-\mathrm{PR}10+1\right)}{\mathrm{PR}11}$$

which shows the proportion of cases due to both exposures; and by the synergy index

$$S=\frac{\left(\mathrm{PR}11-1\right)}{\left(\mathrm{PR}01+\mathrm{PR}10-2\right)}$$

which reflects the direction of interaction in relation to nullity (S = 1), synergy (S > 1) or antagonism (S < 1). The excess of prevalence

EP = P_exposure_ − P_no exposure_

and the excess of prevalence ratio

EPR = PR − 1

were also calculated, which indicate whether the combined effect of the factors is greater than the sum of their effects isolated, and the relative difference

$$\mathrm{RD}=\left[\left(\frac{\mathrm{PR}-1}{\mathrm{EPR}01+\mathrm{EPR}10}\right)-1\right]$$

which shows the departure from the expected behavior for the isolated action of the factors. The formulas were adjusted to three-factor analysis. 

Logistic regression analysis was performed to build a model capable of predicting the association between labor aspects and MS adjusted for potential confounders. Considering epidemiological and literature criteria, the covariates considered in the analysis were the following: age (up to 35 years/over 35 years); practice of physical activities (yes/no) and healthy eating habits (yes/no), all self-reported. The statistical program STATA version 15 was used, licensed by the University of the State of Bahia (UNEB). The measures of PR, 95%CI, and *p* were estimated by the Poisson regression method with robust variance from the odds ratio (OR) values obtained by the logistic regression model.

### 2.7. Ethical Issues

The study was approved by the Ethics Committee for Research involving human beings at UNEB, under opinion no. 872.365/2014. All stages of this study complied with the Helsinki protocol and Resolution 466/12 of the Brazilian Health Council, which deals with the guidelines and regulatory standards for research with human beings. All participants signed an informed consent form.

## 3. Results

The study population consisted of 1125 (94.1% response rate) young nursing professionals (mean age 37.1 years ±9.6), female (87.9%), married (46.1%), black (75.1%), and mainly studied at a technical level (54.4%). In terms of lifestyle, most respondents reported maintaining a diet based on healthy eating (54.0%) and performing physical activities on a regular basis (56.8%). Alcohol consumption was reported by 63.3% of the participants, but it was not statistically associated with MS (PR= 1.09; CI = 0.88–1.35). On the other hand, consumption of tobacco (11.8%) and illegal drugs (1.2%) were associated with the outcome, with emphasis on the latter (PR = 2.57; CI = 1.65–3.99) (data not shown in tables).

The MS prevalence in the studied population was (24.4%) and its evaluation with the characteristics of the work showed statistical significance among professionals with academic education (PR = 1.64; CI = 1.29–2.06); who had income of up to two minimum wages (PR = 1.27; CI = 1.03–1.56); with professional exhaustion (PR = 1.79; CI= 1.44–2.22); who underwent violence at work (PR = 1.24; CI = 1.01–1.53); with working time in the PHC greater than 5 years (PR = 1.40; CI = 1.14–1.72) and no rest break during work activities (PR = 1.30; CI = 1.06–1.60), making up the profile of greatest vulnerability to the occurrence of MS (Table 1).

The global analysis of the MS components showed, in descending order of occurrence: low HDL cholesterol (44.0%), abdominal obesity (41.5%), hypertriglyceridemia (33.4%), hypertension (28.1%) and hyperglycemia (7.5%). When evaluated by exposure factors, the contribution of the variable occupation was evident, which interfered with the occurrence of all components, especially in the abdominal circumference (PR = 1.26; CI = 1.13–1.67), the working time exerted an influence mainly in blood pressure (PR = 1.55; CI = 1.28–1.86), and professional exhaustion in fasting blood glucose (PR = 2.36; CI = 1.54–3.61) (Table 2).

Regarding the MS prevalence according to the exposure factors considered, there was an occurrence of 13.1% in the group with no exposure and 44.9% among those exposed to the three investigated factors. The combination of exposures showed an increasing trend in prevalence ratios, culminating with a PR = 3.42 in the group with the highest exposure (CI = 2.28–5.13). The analysis of the effect of isolated factors showed that burnout exerted a greater influence on the occurrence of the outcome (PR = 1.94; CI = 1.11–3.41) and in the group of combined exposures, there was an increase with greater magnitude of association among those exposed to the factors of working time and professional exhaustion (PR = 3.15; CI = 1.85–5.37). In the combined exposure groups, exposure factors remained statistically associated with the outcome even after adjustment (Table 3).

Considering the additivity assumption, the difference between the expected effect due to the combined exposure (P = 28.9%) and the prevalence observed for the group (P = 44.9%) exceeded the occurrence of the outcome by 16.0%. The measure of the effect association among occupation, burnout, and working time (PR = 3.42) was higher than that estimated based on the additivity of the effects (PR = 2.19), presenting an excess of risk due to exposure of 1.23. The proportion of cases attributed to the interaction was 35.0% with direction towards the synergy of effect (S = 2.03) (Table 3).

The data showed that 13.1% of the MS prevalence corresponded to unknown exposures as it represented individuals who developed the outcome even without being exposed to any of the investigated factors. On the other hand, 12.4% of the prevalence of the outcome was registered in the group of exposure to occupational burnout, 10.8% among those exposed to occupation, and 5.7% to working time. Observing an excesses of prevalence ratios, it was noticed that the combined effect of the factors (EPR = 2.42) was greater than the sum of their isolated effects (EPR = 2.19). The relative difference between the effect estimates indicates that there is a 10.5% departure from the expected behavior for action regardless of the factors (Table 4).

## 4. Discussion

This is the first study that investigates the interactions among occupation, burnout, and working time for the occurrence of MS among PHC nursing professionals. Although there is evidence in the literature of the association between work and MS [4,16,17,18], the scarcity of studies of this nature is evident, especially in the Brazilian context.

The sociodemographic profile of PHC nursing professionals in Bahia corroborates the Brazilian profile: a young population, predominantly female, black, and without academic education [19]. The lifestyle characteristics were compatible with the maintenance of good health; however, the self-reporting of the variables in question may not have reflected the reality experienced, since, considering the population under study and social expectations about the care they should have with their own health, it may have induced more positive responses.

Regarding the MS occurrence, it was lower than in another Brazilian study conducted with nursing professionals [20]; however, its prevalence was relevant and reached one professional out of every five investigated. The association of MS with the occupational variables in this study was based on the results of previously published investigations and is the subject of an increasing number of investigations [4,5,18,21,22].

MS is a complex clinical entity, i.e., the result of a combination of genetic vulnerabilities and environmental exposures. As a syndrome, it requires the simultaneous occurrence of several homeostatic disorders, whose repercussions have significant impacts on the morbidity and mortality profiles of the patients. The literature points to a causal relationship between work aspects and MS, indicating the relevance of work as a risk factor for its occurrence [22,23,24,25].

In this study, it was clear that the combined effect of occupation at a technical level, professional exhaustion, and working time in PHC greater than 5 years produced more cases than the expectation, based on the simple additivity of the effects, evidencing an interaction of a synergistic nature. Thus, it can be said that this scenario is more favorable for the occurrence of MS among PHC nursing professionals in Bahia, Brazil.

Regarding occupation, the characteristics of work today make up vulnerability profiles that expose workers to physical and mental illnesses. In the health sector, physical and psychosocial risk factors are even more striking in a technical level group [26]. In the context of nursing, these professionals receive lower salaries, and repercussions reverberate in the most diverse factors associated with MS, especially regarding access to processed and ultra-processed foods and sedentary lifestyles. It is also worth mentioning that lower incomes can determine the need to assume other employment relationships. In PHC the weekly workload is 40 h per week for the nursing team [27], which often includes night shifts; there is scientific evidence about the repercussions on MS occurrences [24,28,29].

The impact of lifestyle on metabolic outcomes has been widely discussed in the specialized literature. Diet, physical activity, and sleep habits are a combination of important lifestyle factors for MS occurrence as they interfere in the relationship between consumption and energy expenditure, with consequent insulin resistance and obesity, which are determining factors for MS [30,31].

Furthermore, nursing at a technical level is marked by conducting routine tasks. Scripted and prescriptive tasks, with no room for expression of the worker’s subjectivity, might result in dissatisfaction with the work and, ultimately, MS. The biological mechanisms responsible for the effects of job satisfaction and the risk of developing MS are still poorly understood. One path possibly suggested involves inflammatory markers that are pronounced among dissatisfied individuals, contributing to the risk of metabolic outcomes [32,33]. Another plausible mechanism is associated with secondary obesity and unfavorable health behaviors, such as overeating or sedentary lifestyles adopted by less satisfied people [34].

Professional exhaustion, in turn, adds numerous unfavorable characteristics of work and its occurrence is high among PHC professionals. Considering the labor dynamics of nursing professionals specialized in PHC and the required bond formations with patients, professional exhaustion often occurs and causes numerous negative health repercussions, including MS, in nursing workers [8].

The literature shows that professional exhaustion mobilizes the hypothalamic–pituitary–adrenal axis, leading to hypercortisolism (and its resulting aspects) as a result. Visceral obesity and insulin resistance are core factors for MS, since an imbalance of the autonomic nervous system and elevated catecholamines develop in a highly favorable biomolecular environment for cardiometabolic diseases. However, in terms of causal mechanisms, there is still a gap in the literature as to which biological marker is capable of defining professional exhaustion, making it difficult to establish the marker’s contribution to negative repercussions on health [35].

In addition, professional exhaustion predisposes individuals to adopt less healthy lifestyle habits, e.g., consumption of more caloric foods, increased frequency of meals, tobacco consumption, alcohol, and illicit substance abuse, which can cause an increase in MS occurrences [36,37,38].

Still, concerning the factors investigated in this study, the analyses showed that working time was associated with the outcome, increasing its magnitude in the combined exposure groups, whose synergistic interactions were evidenced in the exposure group to the three factors, even after adjusting the variable ‘age’. This finding can be explained by the fact that chronic exposure to work stressors in PHC is imperative in the occurrence of the outcome. Perhaps the length of work itself is not a risk factor for illness, but the way the work context presents itself over time might promote certain illness profiles among workers and health professionals and professional burnout as a result.

Professional exhaustion occurs due to chronic stress to which the individual is subjected in daily work, making the role of exposure time to stressful factors understandable and, thus, depleting the compensatory resources responsible for maintaining homeostasis. In PHC, some aspects of work have already been listed as stressors and contributors to the occurrence of professional exhaustion, namely greater workloads, length of service, low pay, dissatisfaction with work, lack of rest breaks, and unfavorable work conditions [9,39]. Therefore, exposure to these factors (over the years) is a risk factor for workers and MS.

It should be noted that the design of this study did not allow for establishing causal relationships and it has limitations. Information on exposure variables was the result of self-reports, which provided subjective data and were, therefore, susceptible to bias. There was also the possibility of a healthy worker bias since the study only included individuals who were working without restrictions about their professional activities. Those who were already ill did not participate in the sample studied. Furthermore, there may have been limitations in generalizing the results to other populations with different occupations, as all participants in this study were from a specific professional class. However, it is worth noting that the careful statistical treatments of the data and the robustness of the analyses performed allowed us to safely infer the relationships between the investigated exposure factors and MS. Furthermore, the sample in this study was representative of nursing professionals from a large Brazilian state, allowing the findings to be projected to similar populations.

## 5. Conclusions

The findings of the study allowed us to evidence the existence of synergistic interaction between work and MS among PHC nursing professionals; that is, professionals at a technical level, with professional exhaustion, and more than five years of experience in PHC, were the most vulnerable group to MS. The literature points to the biological plausibility of the associations found here. It is important to provide interventions in workplaces where these professionals work in order to reduce the number of cases, whose repercussions will have impacts on the morbidity and mortality statistics of chronic non-communicable diseases. Other studies should be developed with the objective of estimating the causality between work variables and metabolic syndrome.

## Figures and Tables

**Table 1:** Work characteristics according to the occurrence of metabolic syndrome in the studied population, Bahia, Brazil, 2018.

Variables (N)	n(%)	Metabolic Syndrome		
		No n(%)	Yes n(%)	PR (95%CI)
**Occupation (n = 1125)**				
Nurses	452 (40.4)	372 (82.3)	80 (17.7)	1.00
Nursing Assistants/technicians	659 (59.6)	468 (71.0)	191 (29.0)	1.64 (1.29–2.06)
**Monthly income (n = 1125)**				
Two minimum wages	512 (46.5)	371 (72.5)	141 (27.5)	1.27 (1.03–1.56)
Three or more minimum wages	599 (53.5)	469 (78.3)	130 (21.7)	1.00
**Professional burnout (n = 1121)**				
No	902 (81.7)	710 (78.7)	192 (21.3)	1.00
Yes	205 (18.3)	127 (62.0)	78 (38.0)	1.79 (1.44–2.22)
**Night shift (n = 1125)**				
No	884 (79.5)	678 (76.7)	206 (23.3)	1.00
Yes	227 (20.5)	162 (71.4)	65 (28.6)	1.23 (0.97–1.56)
**Working conditions (n = 1125)**				
Satisfactory	686 (62.0)	524 (76.4)	162 (23.6)	1.00
Unfavorable	425 (38.0)	316 (74.4)	109 (25.6)	1.09 (0.88–1.34)
**Double employment relationship (n = 1125)**				
No	780 (70.2)	594 (76.2)	186 (23.8)	1.00
Yes	331 (29.8)	246 (74.3)	85 (25.7)	1.08 (0.86–1.34)
**Violence at work (n = 1125)**				
No	740 (66.8)	573 (77.4)	167 (22.6)	1.00
Yes	371 (33.2)	267 (72.0)	104 (28.0)	1.24 (1.01–1.53)
**Working time at APS (n = 1125)**				
Up to 5 years	642 (57.4)	508 (79.1)	134 (20.9)	1.00
More than 5 years	469 (42.6)	332 (70.8)	137 (29.2)	1.40 (1.14–1.72)
**Employment relationship (n = 1125)**				
not precarious	853 (77.0)	633 (74.2)	220 (25.8)	1.00
precarious	258 (23.0)	207 (80.2)	51 (19.8)	0.77 (0.58–1.00)
**Satisfaction with work (n = 1125)**				
Yes	974 (87.7)	741 (76.1)	233 (23.9)	1.00
No	137 (12.3)	99 (72.3)	38 (27.7)	1.16 (0.87–1.55)
**Rest break (n = 1125)**				
Yes	658 (59.7)	515 (78.3)	143 (21.7)	1.00
No	453 (40.3)	325 (71.7)	128 (28.3)	1.30 (1.06–1.60)

**Table 2:** Work characteristics according to the occurrence of the clinical parameters of metabolic syndrome in the population studied, Bahia, Brazil, 2018.

Variables	Blood Pressure		HDL Cholesterol		Triglycerides		Fasting Glucose		Abdominal Circumference	
	P	PR (p)	P	PR (p)	P	PR (p)	P	PR (p)	P	PR (p)
**Occupation**										
Nurses	21.8	1.00	40.5	1.00	28.8	1.00	5.3	1.00	33.0	1.00
Assistants/technicians	32.4	1.22 (≤0.01)	46.4	1.10 (0.02)	36.6	1.15 (≤0.01)	9.0	1.21 (0.01)	47.3	1.26 (≤0.01)
**Working time**										
Up to 5 years	22.8	1.00	42.7	1.00	32.6	1.00	7.2	1.00	35.8	1.00
More than 5 years	35.3	1.55 (≤0.01)	45.8	1.07 (0.14)	34.5	1.06 (0.24)	7.9	1.10 (0.32)	49.3	1.37 (≤0.01)
**Professional burnout**										
No	24.5	1.00	44.7	1.00	31.1	1.00	6.0	1.00	40.5	1.00
Yes	44.4	1.82 (≤0.01)	41.5	0.93 (0.20)	43.4	1.39 (≤0.001)	14.1	2.36 (≤0.001)	45.4	1.12 (0.10)

**Table 3:** Prevalence and prevalence ratios of isolated and combined exposures according to the occurrence of MS.

				Metabolic Syndrome				
Exposure Variables	N	P	PR	CI95%	p	ILO	CI95%	p
**Reference**								
Occupation = 0, professional burnout = 0, working time = 0	267	13.1	1.00			1.00		
**isolated exhibitions**								
Occupation = 0, professional burnout = 0, working time = 1	101	18.8	1.43	0.86–2.38	0.08	1.27	0.82–1.99	0.27
Occupation = 1, professional burnout = 0, working time = 0	264	23.9	1.82	1.24–2.65	0.01	1.36	0.95–1.94	0.08
Occupation = 0, professional burnout = 1, working time = 0	51	25.5	1.94	1.11–3.41	0.01	1.30	0.73–2.32	0.36
**Combined exhibitions**								
Occupation = 1, professional burnout = 0, working time = 1	270	27.8	2.11	1.47–3.04	0.01	1.51	1.06–2.15	0.02
Occupation = 1, professional burnout = 1, working time = 0	56	39.3	2.99	1.91–4.69	0.01	2.51	1.65–3.80	0.01
Occupation = 0, professional burnout = 1, working time = 1	29	41.4	3.16	1.85–5.37	0.01	2.92	1.93–4.41	0.01
Occupation = 1, professional burnout = 1, working time = 1	69	44.9	3.42	2.28–5.13	0.01	2.37	1.61–3.49	0.01
Expected additive effect ^a^		28.9						
Expected prevalence ratio ^b^			2.19					
s ^c^			2.03					
RERI ^d^			1.23					
AP ^and^			0.35					

RPa: diet, physical activity, and age-adjusted prevalence ratio. ^a^ Expected additive effect = P001 − P000 + P010 − P000 + P100 − P000. ^b^ Expected prevalence ratio = RP001 − RP000 + RP010 − RP000 + RP100 − RP000. ^c^ Synergy index (S) = (RP11 − 1)/(RP01 + RP10 − 2). ^d^ Excessive risk due to exposure (RERI) = RP11 − RP01 − RP10 + 1. ^and^ Proportion of cases attributed to the interaction (AP) = (RP11 − RP01 − RP10 + 1)/RP11.

**Table 4:** Prevalence excesses and prevalence ratios for isolated and combined effects of occupation, professional exhaustion, and working time on the occurrence of metabolic syndrome.

Variable	Over Prevalence ^A^	Excess Prevalence Ratio (EPR = PR − 1)		Relative Difference A/B (1)
		Observed (EPRO) (A)	Expected Based on Separate Exposures ^B^ (B)	
**Reference**				
Occupation = 0, professional burnout = 0, working time = 0	-		-	
**Isolated exhibitions**				
Occupation = 1, professional burnout = 0, working time = 0	10.8	0.82	-	
Occupation = 0, professional burnout = 1, working time = 0	12.4	0.94	-	
Occupation = 0, professional burnout = 0, working time = 1	5.7	0.43	-	
**Combined exhibitions**				
Occupation = 0, professional burnout = 1, working time = 1	28.3	2.16	1.37	57.6
Occupation = 1, professional burnout = 1, working time = 0	26.2	1.99	1.76	13.0
Occupation = 1, professional burnout = 0, working time = 1	14.7	1.11	1.25	−11.2
Occupation = 1, professional burnout = 1, working time = 1	31.8	2.42	2.19	10.5

^A^ Over-prevalence = PR _exposure_ − PR _no exposure_). ^B^ Expected excess prevalence ratio based on separate exposures = EPRO _01_ + EPRO _10._
